# CTA-Based Truncal-Type Occlusion Is Best Matched With Postprocedural Fixed Focal Stenosis in Vertebrobasilar Occlusions

**DOI:** 10.3389/fneur.2018.01195

**Published:** 2019-01-21

**Authors:** Seong-Joon Lee, Ji Man Hong, Jin Wook Choi, Dong-Hun Kang, Yong-Won Kim, Yong-Sun Kim, Jeong-Ho Hong, Joonsang Yoo, Chang-Hyun Kim, Sung-Il Sohn, Yang-Ha Hwang, Jin Soo Lee

**Affiliations:** ^1^Department of Neurology, Ajou University Medical Center, Ajou University School of Medicine, Suwon, South Korea; ^2^Department of Radiology, Ajou University Medical Center, Ajou University School of Medicine, Suwon, South Korea; ^3^Department of Neurosurgery, School of Medicine, Kyungpook National University, Daegu, South Korea; ^4^Department of Radiology, School of Medicine, Kyungpook National University, Daegu, South Korea; ^5^Department of Neurology, School of Medicine, Kyungpook National University, Daegu, South Korea; ^6^Department of Neurology, Keimyung University Dongsan Medical Center, Daegu, South Korea; ^7^Department of Neurosurgery, Keimyung University Dongsan Medical Center, Daegu, South Korea

**Keywords:** endovascular treatment, computed tomographic angiography, intracranial atherosclerotic stenosis, truncal-type occlusion, intracranial atherosclerosis

## Abstract

**Background:** Differentiation of embolic and atherosclerotic occlusions is difficult prior to endovascular treatment (EVT) of acute ischemic stroke due to intracranial large artery occlusions. CTA-determined occlusion type has been reported to be associated with a negative cardiac embolic source and stent retriever failure, a potential of intracranial atherosclerosis (ICAS)-related occlusions. In this study, we evaluated the agreement between preprocedural identification of CTA-determined truncal-type occlusion (TTO) and postprocedural evaluation of underlying fixed focal stenosis (FFS) in the occlusion site.

**Methods:** Patients who underwent intracranial EVT for acute ischemic stroke within 24 h of onset and who had baseline CTA were identified from a multicenter registry collected between January 2011 and May 2016. Preprocedural occlusion type was classified as TTO (target artery bifurcation saved) or branching-site occlusion (bifurcation involved) on CTA. As for postprocedural identification, FFS was evaluated by stepwise analyses of procedural and postprocedural angiographies. The agreement between TTO and FFS was evaluated in respective intracranial vascular beds. Receiver operating characteristics analyses were also performed.

**Results:** A total of 509 patients were included [intracranial internal carotid artery (ICA): 193, middle cerebral artery (MCA) M1: 256, and vertebrobasilar artery (VBA): 60]. In preprocedural identification, 33 (17.1%), 41 (16.0%), and 29 patients (48.3%) had TTOs, respectively. TTOs had good agreement with angiographic FFS in M1 (positive predictive value: 63.4%, negative predictive value: 83.2%, likelihood ratio: 5.42, *P*_multivariate_ < 0.001) and VBA (72.4%, 96.8%, and 4.54, respectively, *P*_multivariate_ = 0.004), but not in intracranial ICA occlusions (*P*_multivariate_ = 0.358). The area under the receiver operating characteristics curve was the largest for VBA (0.872, *p* < 0.001), followed by MCA M1 (0.671, *p* < 0.001), and intracranial ICA (0.551, *p* = 0.465).

**Conclusions:** Agreement between preprocedural TTO and postprocedural FFS, both of which are surrogate markers for ICAS-related occlusions, is highest for VBA, followed by MCA M1 occlusions. There is no significant association in intracranial ICA.

## Introduction

The recent success of several randomized controlled trials of endovascular treatment (EVT) (1–7) has resulted in adoption of EVT as standard therapy for acute stroke due to intracranial large artery occlusion. Contemporary endovascular therapy focuses on removal of intraluminal emboli through stent retrieval or direct aspiration (8–11). Nevertheless, a substantial fraction of intracranial occlusions are possibly caused by intracranial atherosclerotic stenosis (ICAS)-related occlusions, especially in the Asian population (12–17). Although EVT for ICAS-related occlusion is reported to be as safe and effective as for embolic occlusions (13, 16, 18, 19), treatment refractoriness and reocclusion (15, 20) during the procedure is frequently reported, resulting in procedure time elongation and relatively poor prognosis (14, 17). Furthermore, because ICAS-related occlusion cannot be easily differentiated by baseline imaging prior to the procedure, the effectiveness of EVT is difficult to confirm in a randomized prospective trial.

As an etiological approach, ICAS-related occlusions with EVT cannot be confirmed by pathological evaluations. Thus, a definitive method has not been developed for confirming the underlying ICAS in intracranial large artery occlusions either prior to or after the procedure. Instead, surrogate markers have been suggested by several studies. Two surrogate markers for ICAS-related occlusion have been reported: fixed focal stenosis (FFS) and truncal-type occlusion (TTO).

Postprocedural identification of ICAS can be performed by digital subtraction angiography (DSA) images taken during EVT (14). This method focuses on the presence of significant FFS (12), which is revealed during or after the procedure by transfemoral cerebral angiography. Because of its intuitiveness, this method has been used widely in previous studies (13, 19, 21). However, target residual stenosis may not always be the culprit underlying the occlusion because some thrombotic remnant stenoses can mimic ICAS (20). Intraprocedural complications, including vasospasm or dissections, may also complicate the etiological classification. Therefore, a stepwise approach to diagnose ICAS-related occlusion is necessary, incorporating both procedural DSA and postprocedural repeat non-invasive vascular imaging (17, 22).

An important issue regarding ICAS-related occlusion is its preprocedural identification (23, 24). DSA-determined TTO is another surrogate marker of ICAS-related occlusion that can potentially address this issue. Kim, Baek and colleagues had previously performed impressive studies on occlusion types, TTOs and branching-site occlusions (BSOs), in intracranial large arteries. In 2016, they reported that the DSA-determined TTO type at the time of deployment of the stent retriever during EVT was significantly associated with stent retriever failure (25). Moreover, the TTO type was associated with the absence of embolic sources, which were thoroughly investigated by postprocedural etiological approaches such as echocardiography, cardiac computed tomography (CT), and aortic arch atheroma imaging (25). In 2017, they reported that these occlusion types could be applied to computed tomographic angiography (CTA). Through their internal validation, the BSO type on preprocedural CTA corresponded well to the same type on DSA-based evaluations (26). This dichotomized analysis would be practically useful because it can also be assessed by baseline CTA (26), a time-sparing and widely-used imaging tool in EVT for acute stroke.

We hypothesized that the predictive ability of the preprocedural CTA-based identification of occlusion types may differ among occlusive vascular beds due to variations in their branching locations and in levels of collaterals. Therefore, in this study, we aimed to evaluate the agreement between the TTO, a preprocedural occlusion type, and the FFS, a postprocedural surrogate marker of ICAS-related occlusion, in the three most common occlusion sites targeted by EVT, including the intracranial internal carotid artery (ICA), the middle cerebral artery M1 portion (MCA M1), and the vertebrobasilar artery (VBA), using a retrospective multicenter EVT database.

## Materials and Methods

### The ASIAN KR Registry

The Acute Stroke due to Intracranial Atherosclerotic occlusion and Neurointervention—Korean Retrospective (ASIAN KR) registry is a three-center retrospective database consisting of consecutive patients ages 18 or older who underwent EVT for treatment of acute ischemic stroke due to intracranial and/or extracranial large vessel occlusion (27). The registry focuses on revealing the clinical and procedural characteristics of ICAS-related occlusion, a frequent etiology in Asian population, yet in which interventional outcomes are less well-defined. The data collection protocol was approved by the Institutional Review Board of each hospital and was implemented in accordance with the ethical standards of the 1964 Declaration of Helsinki and its later amendments. All EVT procedures were performed at the discretion of the treating physician. The datasets generated in this study are available on reasonable request to the corresponding author. The current study was retrospectively performed with comparative and descriptive analyses based on the ASIAN KR registry.

### Core Lab Imaging Evaluations

After de-identification and blinding of clinical data, core laboratory imaging analyses were performed to ensure consistent grading and eliminate bias. The location of the initial large vessel occlusion was identified on baseline angiography (S-JL). Alberta Stroke Program Early CT scores (ASPECTS) were classified on non-contrast CT (S-IS). Successful reperfusion was classified as modified Treatment In Cerebral Ischemia (mTICI) grade 2b−3 (JSL, Y-HH) (28). Arterial recanalization was also classified by the Arterial Occlusive Lesion (AOL) score to grade recanalization degree or residual stenosis of target occlusive lesions (JSL, Y-HH) (29). Parenchymal hematoma type 2 (30) and/or modified Fisher scale grade 3–4 subarachnoid hemorrhages (31) were regarded as serious postprocedural hemorrhagic complications (S-IS).

### Preprocedural Identification of Truncal-Type Occlusion

Based on baseline CTA, occlusion types were classified as either TTO or BSO according to previous reports (25, 26), by two individual interventional neurologists (S-JL, 4 years of clinical experience; JY, 5 years of clinical experience). For the current study, it was classified as TTO if the very next bifurcation site was clearly visible beyond the occlusion segment. Occlusion types that involved the bifurcation sites of the vascular beds and/or major branches were classified as BSOs. Cases in which CTA images were not of sufficient quality to differentiate between the two occlusion types were classified as inconclusive and were excluded from the analysis. For the MCA M1, if the bifurcation point and most proximal point of both M2 segments were visible, it was regarded as a TTO. In intracranial ICA occlusions, a T-type occlusion that involves both the ICA bifurcation point, M1 proximal, and A1 portion was classified as BSO, while an I-type occlusion with occlusion of only the distal ICA with visualization of the proximal anterior cerebral artery (ACA) A1 and MCA junction through collaterals was classified as TTO. If the top of the basilar artery was involved in a basilar artery occlusion, it was regarded as a BSO. By contrast, if the top was saved, it was regarded as a TTO. Likewise, the basilar bifurcation was also evaluated in the case of dominant V4 occlusions resulting in major posterior circulation syndromes.

The CTA was obtained according to the protocol of each hospital. At center A, the CT scans (SOMATOM Sensation 16; SOMATOM Definition Edge [128-channel] Siemens, Erlangen, Germany), including non-contrast and postcontrast axial parenchymal images, were acquired with contiguous 5-mm thick axial sections (120 kV, 270 mAs). For CTA, a maximum of 90 mL (1.2 mL/kg) of iodinated contrast agent was injected at 4 mL/s, immediately followed by a 15 mL saline bolus. A bolus tracking technique was used with a minimum delay of 3-s. CTA images were acquired from the aortic arch to the vertex with the following parameters: 100 kV, 180 mAs, 0.5 s per rotation, 0.5 pitch, and a 0.75-mm section thickness. The CT source images were postprocessed to create coronal, sagittal, and axial multiplanar reformats in maximum intensity projection (MIP) images (10-mm slab and 2-mm interval) and volume-rendered 3D images.

At center B, (Optima CT 660 [64-channel]; Revolution EVO [128-channel], GE, Boston, MA) non-contrast CT was acquired with 2.5-mm thickness and 2.5-mm interval (tube voltage of 120 kV). CT angiography was performed using 2.5-mm slice thickness, 2.5-mm reconstruction interval, 100 kV, 100–300 mAs. A maximum of 80 mL (2 mL/kg) of iodinated contrast agent was injected at 4 mL/s, immediately followed by a 50 mL saline bolus. The CT source images were postprocessed to create coronal, sagittal, and axial multiplanar reformats in MIP images (10-mm slab and 2-mm interval) and volume-rendered 3D images. MIP images were obtained after May 2012 at center B.

At center C (SOMATOM Definition Flash [128-channel], Siemens, Erlangen, Germany), non-contrast CT was taken with 5-mm thickness and 5-mm interval (tube voltage of 120 kV). The first phase of the multiphase CTA is from the aortic arch to the vertex using a multidetector CT scanner, acquired in the late arterial phase with scanning triggered by contrast bolus monitoring in the aortic arch with average dose length product of 700–800 mGy cm. The scan time is < 10 s. Images were acquired with a 0.625-mm section thickness. The second phase was acquired after a delay of 4 s that allows for table repositioning to the skull base. The scanning duration for each additional phase was 3.4 s. Thus, the 3 phases were each 8 s apart. A total of 70 mL of contrast material was injected at a rate of 5 mL/s, and was followed by a 50-mL normal saline chase at a rate of 4 mL/s. (FOV 200^*^200, 100–300 mAs). The CT source images were postprocessed to create coronal, sagittal, and axial multiplanar reformats in MIP images (24-mm slab and 4-mm interval) and volume-rendered 3D images. MIP images and multiphase CT was performed after June 2014 at center C.

### Postprocedural Identification of FFS

An FFS was identified by several steps of angiographical analysis and was evaluated and determined based on the consensus of a vascular neurologist (Y-HH, over 10 years of clinical experience) and an interventional neurologist (JSL, 10 years of clinical experience) (14, 22): (1) uncommon stroke etiologies such as dissection, Moyamoya disease, and vasculitis were evaluated by DSA performed just prior to EVT. (2) If the occluded vessel was completely recanalized after primary thrombectomy, the etiology was determined to be embolic occlusion. (3) A focal stenosis >70% or lower-degree stenosis with tendency of re-occlusion or flow impairment during the procedure was defined as a meaningful FFS. (4) Repeat DSAs were often performed up to 20 min after final recanalization, to evaluate residual stenosis, further recanalization, or reocclusion. (5) Any postprocedural non-invasive angiographies performed (CT or MR) were reviewed to evaluate possible changes to the classification (JY).

**Figure 1:**
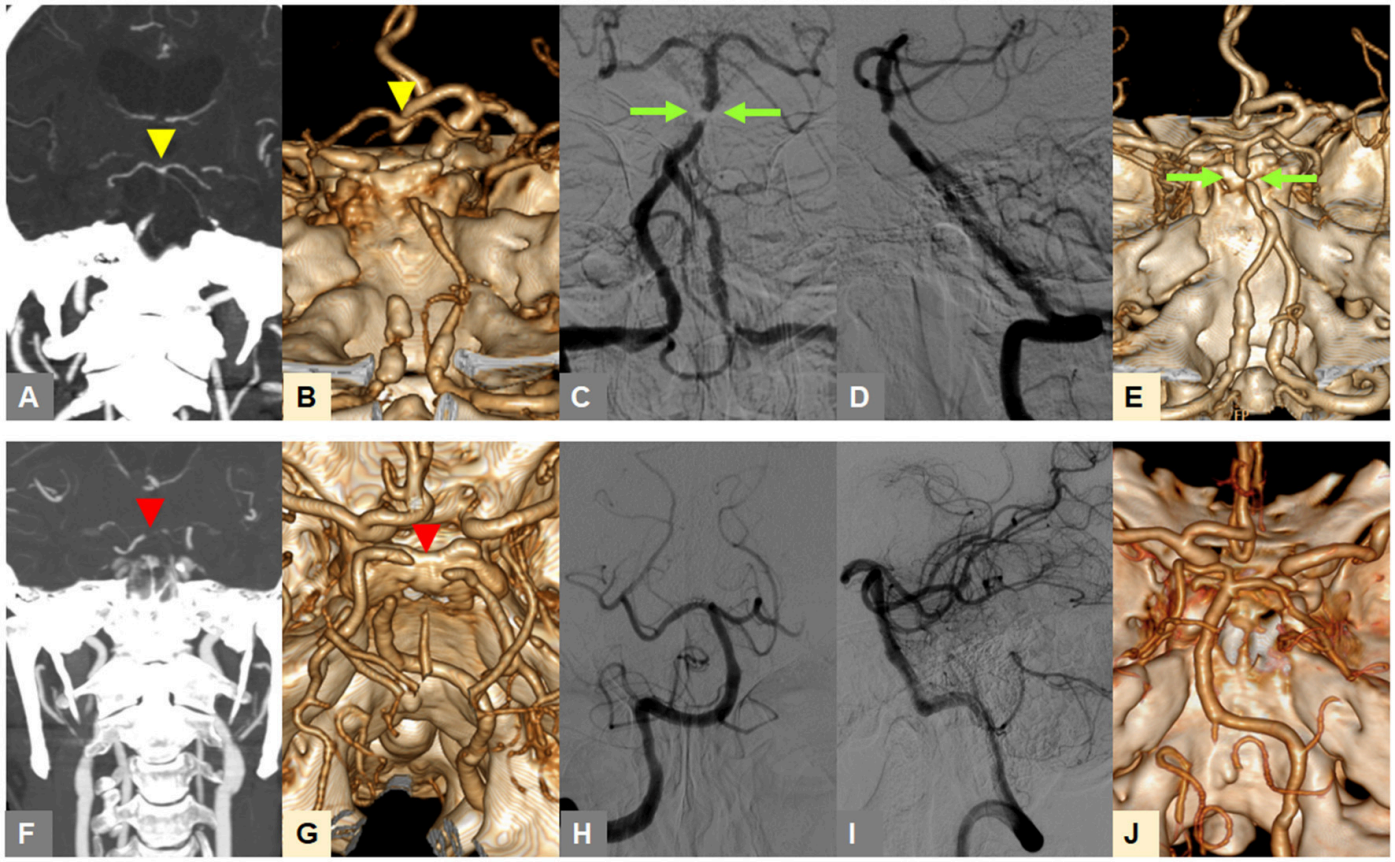
Representative cases for classification of occlusion types on baseline CTA and angiography-revealed fixed focal stenosis. **(A,B)** A truncal-type occlusion of the basilar artery, with clearly visible bifurcation point at the top of the basilar artery (yellow arrowheads) is observed on baseline CTA. **(C,D)** Digital subtraction angiography after reperfusion treatment reveals >70% residual stenosis (green arrow). **(E)** Post-procedure CTA shows underlying atherosclerotic lesion (green arrow). **(F,G)** A branch-site occlusion of the basilar artery with involvement of the top of the basilar portion and P1 segments (red arrowheads) is observed. **(H,I)** Complete recanalization after primary thrombectomy is seen, suggestive of embolic etiology. **(J)** Post-procedure CTA also shows complete recanalization. CTA, computed tomographic angiography.

In our preliminary analysis, the tentative identification of FFS until the 4th step was changed into embolism in 0.4% of included patients at the 5th step, and embolism into FFS in 1.9% (17). Figure 1 shows two example cases that presented with TTO (upper row) and BSO (lower row) types on CTA, and were, respectively, classified as FFS and embolic occlusion through stepwise angiographic analysis. If no recanalization could be achieved during the procedure to determine the etiology, it was specifically classified as intractable occlusion. If a FFS was present, the detailed location with the most severe stenosis in the occlusive vessel was further evaluated (i.e., proximal, middle, or distal segment).

### Inclusion and Exclusion Criteria

For the current study, the following criteria were applied: (1) occlusion in the intracranial ICA portion, the MCA M1 segment, or the vertebrobasilar artery were included, while M2 or distal locations were excluded. Patients were also included when (2) onset-to-puncture time was within 24 h, and when (3) baseline angiographic evaluation was performed by CTA. Sole extracranial targets treated were excluded while tandem occlusions were included.

### Statistical Analysis

Clinical characteristics, endovascular findings, and procedures were compared between patients with TTOs and BSOs at three respective occlusion sites, the intracranial ICA, MCA M1, and VBA. Differences between the groups were analyzed using χ^2^ tests for categorical variables and Student's *t*-tests for continuous variables. For each occlusive vascular bed, the sensitivity, specificity, positive predictive value, negative predictive value, and accuracy of preprocedural identification of TTO and postprocedural identification of FFS were investigated through univariate and multivariate analysis. Receiver operating characteristics (ROC) curve analyses of the association of TTO with FFS were performed for the three respective occlusive sites. The impact of the detailed location (i.e., proximal, middle, or distal segment) regarding the FFS on CTA occlusion types was further evaluated according to each vascular bed. A *p* < 0.05 was considered to be significant. Statistical analysis was performed using the SPSS statistical package (version 22.0; SPSS Inc., Chicago, IL).

## Results

A total of 525 patients were included. After excluding 16 patients due to inconclusive CTA, 509 were included in the final analysis (ICA T: 193, MCA M1: 256, and VBA: 60). In the included patients, the CTAs consisted of single-phase CTA MIP images + reconstruction images in 356 (69.9%), multiphase CTA MIP images + reconstruction images in 71 (13.9%), and CTA-enhanced images + reconstruction images in 82 (16.1%). Of these, 33 (17.1%), 41 (16.0%), and 29 patients (48.3%) demonstrated TTOs on baseline CTA in the ICA, MCA, and VBA, respectively. A total of 406/509 (79.8%) patients were classified as BSOs. The overall k-value of interrater reliability was 0.844. The k-value for intracranial ICA was 0.929, 0.727 for MCA M1, and 0.933 for VBA.

Among the occlusion sites, the statistical significance between the preprocedural occlusion types and the angiographic analyses of etiology was somewhat different. Table 1 shows the comparison between TTOs and BSOs in the intracranial ICA occlusions. TTOs were significantly associated with lower NIHSS scores and higher ASPECTS scores. However, there were no significant differences in the angiographic analysis of etiology (*p* = 0.097). Table 2 shows the comparison between TTOs and BSOs in the MCA M1 portion occlusions. There were fewer patients with atrial fibrillation and coronary artery obstructive disease in the TTO group, while smoking was more common. During the procedure, reocclusion after primary reperfusion treatment was more frequent (31.6 vs. 8.5%, *p* < 0.001) and adjunctive treatments, such as intra-arterial tirofiban or angioplasty, were more frequently used. In the angiographic analysis of etiology, TTO was significantly associated with fewer complete recanalization and more frequently revealed a FFS (*p* < 0.001). Table 3 shows the comparison between TTOs and BSOs in the VBA occlusions. There were no significant differences in clinical characteristics. During the procedure, reocclusion after primary reperfusion treatment was more frequent (42.9 vs. 3.2%, *p* < 0.001) and adjunctive treatments were more frequently needed in the TTO group. In the angiographic analysis of etiology, TTO was associated with fewer complete recanalizations and more frequently revealed a FFS (*p* < 0.001).

**Table 1:** Comparison of clinical characteristics and endovascular procedure findings according to preprocedural occlusion types in intracranial internal carotid artery occlusions.

	**TTO (*n* = 33)**	**BSO (*n* = 160)**	***p*-value**
**CLINICAL CHARACTERISTICS**			
Age, years	71 ± 11	68 ± 13	0.183
Male sex	22 (66.7%)	73 (45.6%)	0.028
Diabetes mellitus	10 (30.3%)	46 (28.7%)	0.858
Hypertension	21 (63.6%)	99 (61.9%)	0.849
Atrial fibrillation	18 (54.5%)	93 (58.1%)	0.705
CAOD	5 (15.2%)	22 (13.8%)	0.833
Hypercholesterolemia	11 (33.3%)	44 (27.5%)	0.499
Smoking	8 (24.2%)	31 (19.4%)	0.526
Admission NIHSS score, median [IQR]	15.0 [11.5–19.0]	18.0 [15.0–21.0]	0.002
ASPECTS, median [IQR]	9.0 [8.0–10.0]	5.5 [3.0–8.0]	< 0.001
**REPERFUSION TREATMENT**			
IV thrombolysis	18 (54.5%)	92 (57.5%)	0.755
Onset-to-puncture time (min)	353 ± 220	303 ± 214	0.222
Procedure time (min)	83 ± 63	77 ± 48	0.502
Reocclusion after primary treatment	1 (3.7%)	8 (5.2%)	0.742
Tirofiban infusion	4 (12.1%)	12 (7.5%)	0.381
Intracranial balloon	1 (3.0%)	3 (1.9%)	0.671
Intracranial stenting	0 (0.0%)	16 (10.0%)	0.058
Number of techniques	2.0 [1.0–3.0]	2.0 [1.0–3.0]	0.620
Final AOL grade			0.109
0	4 (12.9%)	11 (6.9%)	
1	0 (0.0%)	6 (3.8%)	
2	5 (16.1%)	10 (6.3%)	
3	22 (71.0%)	133 (83.1%)	
Successful reperfusion	29 (87.9%)	123 (76.9%)	0.159
Post-procedure reocclusion	1/27 (3.7%)	3/123 (2.4%)	0.712
**ANGIOGRAPHIC ANALYSIS OF ETIOLOGY**			**0.097**
Complete recanalization	25 (75.8%)	137 (85.6%)	
Significant fixed focal stenosis	5 (15.2%)	14 (8.8%)	
Intractable	2 (6.1%)	9 (5.6%)	
Others	1 (3.0%)	0 (0.0%)	
**OUTCOMES**			
Serious hemorrhagic complications	2 (6.1%)	32 (20.0%)	0.056
3-month mRS 0–2	17 (51.5%)	67 (41.9%)	0.309

*The data are presented as the mean ± standard deviation, number (%), or median [interquartile range]. TTO, truncal-type occlusion; BSO, branching-site occlusion; CAOD, coronary artery obstructive disease; NIHSS, National Institutes of Health Stroke Scale; ASPECTS, Alberta Stroke Program Early CT score; IV, intravenous; AOL, arterial occlusive lesion; mRS, modified Rankin Scale*.

**Table 2:** Comparison of clinical characteristics and endovascular procedure findings according to preprocedural occlusion types in middle cerebral artery M1 occlusions.

	**TTO (*n* = 41)**	**BSO (*n* = 215)**	***p*-value**
**CLINICAL CHARACTERISTICS**			
Age, years	62 ± 12	66 ± 13	0.074
Male sex	23 (56.1%)	122 (56.7%)	0.939
Diabetes mellitus	11 (26.8%)	59 (27.4%)	0.936
Hypertension	21 (51.2%)	131 (60.9%)	0.246
Atrial fibrillation	9 (22.0%)	104 (48.4%)	0.002
CAOD	0 (0.0%)	21 (9.8%)	0.037
Hypercholesterolemia	7 (17.1%)	57 (26.5%)	0.201
Smoking	17 (41.5%)	42 (19.5%)	0.002
Admission NIHSS score, median [IQR]	14.0 [8.5–20.5]	16.0 [12.0–20.0]	0.734
ASPECTS, median [IQR]	6.5 [4.0–9.0]	7.0 [5.0–9.0]	0.573
**REPERFUSION TREATMENT**			
IV thrombolysis	21 (51.2%)	113 (52.6%)	0.875
Onset-to-puncture time (min)	386 ± 258	328 ± 233	0.157
Procedure time (min)	75 ± 33	69 ± 42	0.381
Reocclusion after primary treatment	12 (31.6%)	18 (8.5%)	< 0.001
Tirofiban infusion	19 (46.3%)	22 (10.2%)	< 0.001
Intracranial balloon	3 (7.3%)	3 (1.4%)	0.022
Intracranial stenting	2 (4.9%)	7 (3.3%)	0.605
Number of techniques	2.0 [1.0–2.0]	1.0 [1.0–2.0]	0.001
Final AOL grade			< 0.001
0	6 (14.6%)	15 (7.0%)	
1	9 (22.0%)	16 (7.5%)	
2	16 (39.0%)	37 (17.3%)	
3	10 (24.4%)	146 (68.2%)	
Successful reperfusion	30 (73.2%)	160 (74.4%)	0.867
Post-procedure reocclusion	2/38 (5.3%)	13/191 (6.8%)	0.725
**ANGIOGRAPHIC ANALYSIS OF ETIOLOGY**			** < 0.001**
Complete recanalization	13 (31.7%)	163 (75.8%)	
Significant fixed focal stenosis	26 (63.4%)	36 (16.7%)	
Intractable	2 (4.9%)	16 (7.4%)	
**OUTCOMES**			
Serious hemorrhagic complications	3 (7.3%)	12 (5.6%)	0.665
3-month mRS 0–2	24 (58.5%)	114 (53.3%)	0.535

*The data are presented as the mean ± standard deviation, number (%), or median [interquartile range]. TTO, truncal-type occlusion; BSO, branching-site occlusion; CAOD, coronary artery obstructive disease; NIHSS, National Institutes of Health Stroke Scale; ASPECTS, Alberta Stroke Program Early CT score; IV, intravenous; AOL, arterial occlusive lesion; mRS, modified Rankin Scale*.

**Table 3:** Comparison of clinical characteristics and endovascular procedure findings according to preprocedural occlusion types in vertebrobasilar artery occlusions.

	**TTO (*n* = 29)**	**BSO (*n* = 31)**	***p*-value**
**CLINICAL CHARACTERISTICS**			
Age, years	67 ± 9	67 ± 10	0.892
Male sex	18 (62.1%)	18 (58.1%)	0.752
Diabetes mellitus	10 (34.5%)	7 (22.6%)	0.307
Hypertension	22 (75.9%)	18 (58.1%)	0.144
Atrial fibrillation	9 (31.0%)	16 (51.6%)	0.106
CAOD	2 (6.9%)	2 (6.5%)	0.945
Hypercholesterolemia	10 (34.5%)	9 (29.0%)	0.650
Smoking	11 (37.9%)	5 (16.1%)	0.056
Admission NIHSS score, median [IQR]	20.0 [13.5–26.5]	19.0 [11.0–27.0]	0.960
**REPERFUSION TREATMENT**			
IV thrombolysis	12 (41.4%)	20 (64.5%)	0.073
Onset-to-puncture time (min)	406 ± 240	349 ± 303	0.427
Procedure time (min)	79 ± 46	62 ± 40	0.143
Reocclusion after primary treatment	12 (42.9%)	1 (3.2%)	< 0.001
Tirofiban infusion	7 (24.1%)	1 (3.2%)	0.017
Intracranial balloon	8 (27.6%)	0 (0.0%)	0.002
Intracranial stenting	3 (10.3%)	2 (6.5%)	0.586
Number of techniques	2.0 [1.0–2.0]	1.0 [1.0–2.0]	0.044
Final AOL			< 0.001
0	2 (6.9%)	5 (16.1%)	
1	2 (6.9%)	1 (3.2%)	
2	17 (58.6%)	0 (0.0%)	
3	8 (27.6%)	25 (80.6%)	
Successful reperfusion	26 (89.7%)	25 (80.6%)	0.329
Post-procedure reocclusion	4/25 (16.0%)	2/26 (7.7%)	0.357
**ANGIOGRAPHIC ANALYSIS OF ETIOLOGY**			** < 0.001**
Complete recanalization	7 (24.1%)	26 (83.9%)	
Significant fixed focal stenosis	21 (72.4%)	1 (3.2%)	
Intractable	1 (3.4%)	3 (9.7%)	
Others	0 (0.0%)	1 (3.2%)	
**OUTCOMES**			
Serious hemorrhagic complications	2 (7.1%)	1 (3.2%)	0.494
3-month mRS 0–2	8 (27.6%)	17 (54.8%)	0.032

*The data are presented as the mean ± standard deviation, number (%), or median [interquartile range]. TTO, truncal-type occlusion; BSO, branching-site occlusion; CAOD, coronary artery obstructive disease; NIHSS, National Institutes of Health Stroke Scale; IV, intravenous; AOL, arterial occlusive lesion; mRS, modified Rankin Scale*.

**Figure 2:**
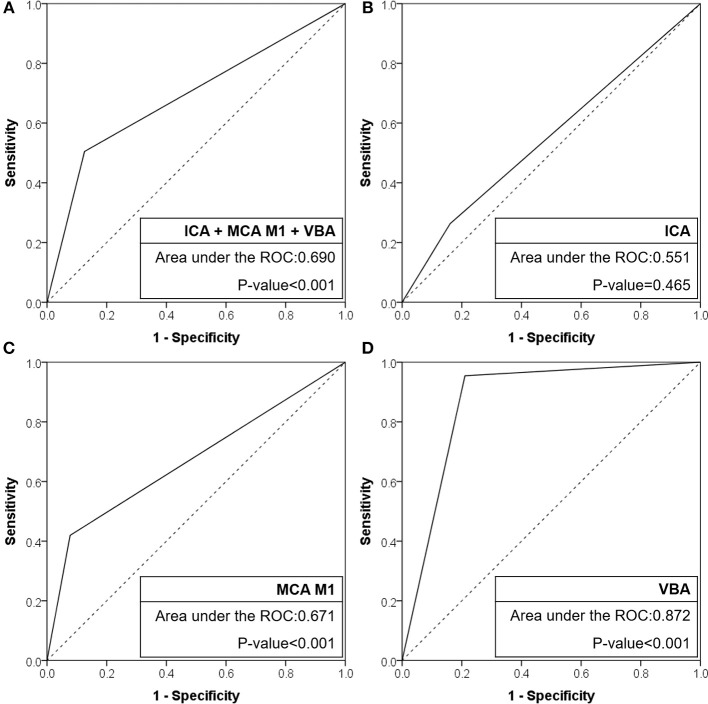
Receiver operating characteristic curves of CTA-based truncal-type occlusions for angiography revealed fixed focal stenosis. **(A)** ICA + MCA M1 + VBA occlusions. **(B)** ICA occlusions. **(C)** MCA M1 occlusions. **(D)** VBA occlusions. CTA, computed tomographic angiography; ICA, internal carotid artery; MCA, middle cerebral artery; VBA, vertebrobasilar artery; ROC, receiver operating characteristic.

When the agreement between TTO and FFS was evaluated (Table 4), the performance was high in overall included vascular beds with moderate sensitivity (50.5%) and high specificity (87.4%) and a positive likelihood ratio of 4.02 (*p* < 0.001). Specifically, the association was significant in multivariate analysis in both MCA M1 (*p* < 0.001) and VBA (*p* = 0.004) occlusions, along with atrial fibrillation as a significant predictor while age, gender, and smoking history were insignificant. By contrast, there was no significance in intracranial ICA occlusion (*p* = 0.358). In the ROC curve analysis (Figure 2), the area under the ROC curve was the largest for the VBA (0.872, *p* < 0.001), followed by the MCA M1 (0.671, *p* < 0.001) and intracranial ICA (0.551, *p* = 0.465).

**Table 4:** The predictive power of CTA-based truncal-type occlusions for angiography-revealed significant fixed focal stenosis, and comparison with initial reports and other imaging modalities.

**References**	**Population**	**Univariate P**	**Multivariate P**	**Sensitivity**	**Specificity**	**PPV**	**NPV**	**Accuracy**	**+LR**	**–LR**
**Truncal-type occlusions in prediction of angiography-revealed significant fixed focal stenosis**										
Current study	ICA + MCA + VBA	<0.001	<0.001^*^	50.5%	87.4%	50.5%	87.4%	80.0%	4.02	0.57
	Intracranial ICA	0.261	0.358^*^	26.3%	83.9%	15.2%	91.3%	78.2%	1.64	0.88
	MCA M1	<0.001	<0.001^*^	41.9%	92.3%	63.4%	83.2%	80.1%	5.42	0.63
	VBA	<0.001	0.004^*^	95.5%	79.0%	72.4%	96.8%	85.0%	4.54	0.06
**CTA truncal-type occlusions in prediction of SR failure**										
Baek et al. (26)	ICA + MCA + VBA			48.2%	90.8%	60.5%	85.6%	81.1%	5.21	0.57
**Truncal-type occlusions after STENT deployment in prediction of SR failure**										
Baek et al. (25)	ICA+MCA+VBA			30.5%	97.6%	–	–	–	12.6	0.71
**Other imaging predictors**										
**Negative susceptibility vessel sign in prediction of ICAS-related occlusion**										
Kim et al. (23)	MCA	<0.001		100.0%	67.1%	42.9%	100.0%	73.6%	3.04	0.00
Suh et al. (24)	ICA+MCA	0.239		28.6%	75.0%	–	–	–	1.14	0.95
**Negative multi-segment clot sign in prediction of large artery atherosclerosis**										
Chen et al. (32)	ICA+MCA	<0.001		90.7%	52.7%	42.9%	93.5%	63.4%	1.92	0.18

* *Multivariate analysis was performed with age, gender, smoking, and atrial fibrillation as covariates. Predictive values from these articles have been calculated through statistical analysis of data presented in the literature. CTA, computed tomographic angiography; PPV, positive predictive value; NPV, negative predictive value; +LR, positive likelihood ratio; –LR, negative likelihood ratio; ICA, internal carotid artery; MCA, middle cerebral artery; VBA, vertebrobasilar artery; SR, stent retriever; ICAS, intracranial atherosclerotic stenosis*.

**Figure 3:**
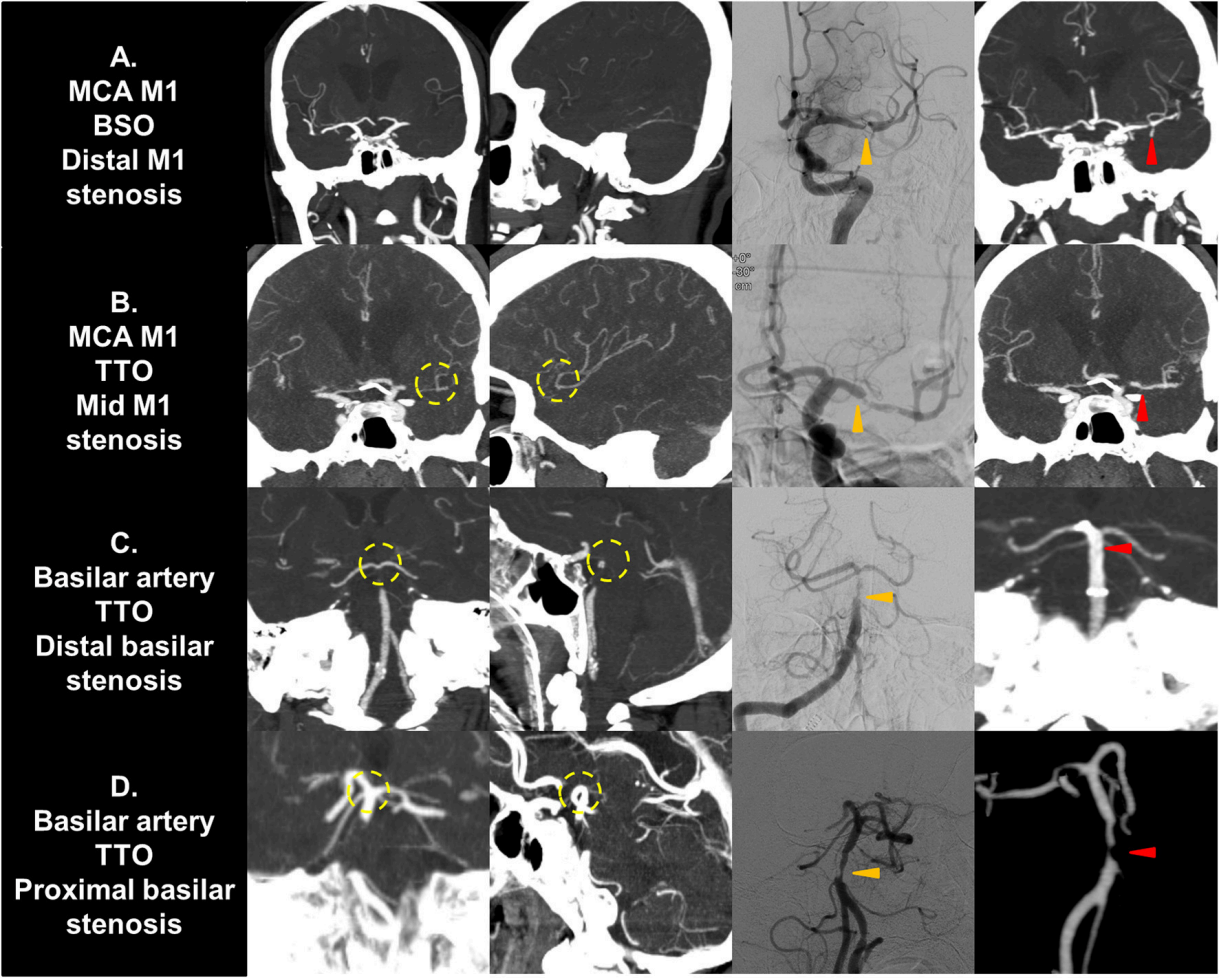
Representative cases of culprit stenotic segment analysis. **(A)** A MCA M1 occlusion with fixed focal stenosis in the distal M1 segment is seen. The shorter distance from culprit stenosis to bifurcation results in a BSO. **(B)** A M1 occlusion with fixed focal stenosis in the mid M1 segment is seen. The distance from stenosis to bifurcation is longer, resulting in a TTO. **(C)** A vertebrobasilar occlusion with fixed focal stenosis in the distal basilar segment is seen. It may present as TTO due to collateral filling from the circle of Willis. **(D)** A vertebrobasilar occlusion with fixed focal stenosis in the proximal basilar segment is seen. The distance from stenosis to bifurcation is longer, resulting in a TTO. Yellow circle, truncal-type occlusions; Orange arrows, fixed focal stenosis in digital subtraction angiography; Red arrows, confirmation in postprocedural non-invasive imaging. MCA, middle cerebral artery; BSO, branch-site occlusion; TTO, truncal-type occlusion.

If a significant FFS was present, a subgroup analysis based on the culprit stenotic segment was performed to identify the impact of the location of FFS on the CTA occlusion type (Table 5). In the intracranial ICA occlusions, a substantial number of culprit stenoses were located in the communicating segment or even the MCA M1 in the BSO group, while the culprit stenosis was more frequently seen in the ophthalmic segment in the TTO group (*p* = 0.026). Overall, underlying M1 FFS was common (57.9%), resulting in frequent BSOs. In the M1 occlusions, stenosis in the proximal or the mid-M1 segment was more frequently seen in the TTO group, while distal M1 or M2 stenosis was more frequently seen in the BSO group (*p* = 0.012). Overall, the stenosis was frequently located in the distal M1 (50.0%), and a significant number of patients presented with BSOs. In VBA occlusions, more proximal involvement was associated with TTOs, while 1 FFS case with distal basilar occlusion showed a BSO (*p* = 0.015). However, distal basilar stenosis was not common, and the majority presented with TTO. Representative cases are seen in Figure 3.

**Table 5:** Comparison of culprit stenotic segments according to occlusion types in each vascular bed in the fixed focal stenosis positive subgroup.

	**Overall**	**TTO**	**BSO**	***P*-value**
**Intracranial ICA occlusions**	***n*** **= 19**	***n*** **= 5**	***n*** **= 14**	**0.026**
Cavernous	1 (5.3%)	0 (0.0%)	1 (7.1%)	
Ophthalmic	5 (26.3%)	4 (80.0%)	1 (7.1%)	
Communicating	2 (10.5%)	0 (0.0%)	2 (14.3%)	
M1 without ACA collaterals	4 (21.1%)	1 (20.0%)	3 (21.4%)	
M1 with ACA collaterals	7 (36.8%)	0 (0.0%)	7 (50.0%)	
**MCA M1 occlusions**	***n*** **= 62**	***n*** **= 26**	***n*** **= 36**	**0.012**
Proximal M1	6 (9.7%)	5 (19.2%)	1 (2.8%)	
Mid M1	23 (37.1%)	13 (50.0%)	10 (27.8%)	
Distal M1	31 (50.0%)	8 (30.8%)	23 (63.9%)	
M2	2 (3.2%)	0 (0.0%)	2 (5.6%)	
**Vertebrobasilar occlusions**	***n*** **= 22**	***n*** **= 21**	***n*** **= 1**	**0.015**
V4 portion	7 (31.8%)	7 (33.3%)	0 (0.0%)	
Proximal basilar	6 (27.3%)	6 (28.6%)	0 (0.0%)	
Mid basilar	7 (31.8%)	7 (33.3%)	0 (0.0%)	
Distal basilar	2 (9.1%)	1 (4.8%)	1 (100.0%)	

*TTO, truncal-type occlusion; BSO, branching-site occlusion; ICA, internal carotid artery; ACA, anterior cerebral artery; MCA, middle cerebral artery*.

## Discussion

The results of the current study show that preprocedural identification of TTO highly agreed with postprocedural identification of FFS, but the degree of agreement differed among occlusion sites. This agreement was the highest in VBA occlusions, followed by M1 occlusions. While the TTO showed insignificant power in intracranial ICA occlusions, it showed moderate sensitivity with higher specificity and positive likelihood ratios in the MCA M1 segment, and both high sensitivity and specificity in the VBA. The area under the ROC curve was the largest in the VBA occlusions followed by MCA M1 occlusions, while the area was insignificant in ICA T occlusions. Although a definitive method for evaluating ICAS-related occlusion was not used, the high agreement between both preprocedural and postprocedural surrogate markers may suggest potentials in practical use.

The agreement between TTO and FFS depended on the vascular bed. This finding may be explained by differences in nearby collateral systems and the frequent location of culprit stenotic segments and their distances from the bifurcation. In VBA occlusions, in which the area under the curve analysis was the largest, culprit segments were predominant in the V4 to the mid-basilar segments, with some distance from the bifurcation. Furthermore, arterial filling distal to the occlusive portion is supplied by both the circle of Willis and leptomeningeal collaterals, and may have resulted in the high sensitivity in the occlusion type analysis. In the MCA, however, the culprit stenotic segment was most frequent in the distal M1, adjacent to the bifurcation point, possibly resulting in a BSO-type occlusion morphology. Furthermore, it is supplied only by leptomeningeal collaterals, which may affect blood stasis and *in situ* thrombosis propagation distal to the culprit ICAS lesion (33) resulting in masking of these occlusions as BSOs rather than visualizing the true stenotic segment. In ICA occlusions, a substantial number of culprit segments were located in the M1 segment in reality, with thrombus propagation proximally and impaired filling of the distal ICA. Furthermore, tortuous cavernous ICA segments can be prone to occlusions due to large emboli (34). For these reasons, both sensitivity and specificity might be unsatisfactory in this group, and the predictive power was insignificant.

Some differences in the methodology for evaluating CTA-based identification of TTO and BSO between the current study and the initial reports might also have affected the predictive power. First, tandem occlusions were included in this study, while their inclusion is uncertain in the original reports. In ICA occlusions, proximal ICA occlusions and ICA-I type occlusions are difficult to differentiate through CT images in some cases, while both may show TTOs, complicating the etiologic analysis of intracranial targets. Accordingly, sole extracranial targets were excluded in a retrospective manner in the current study. Second, TTO was not defined in detail for the vertebrobasilar junction occlusions in the initial reports; therefore, we further defined this situation. Third, while intractable cases were included in the dichotomized analysis in the initial reports, they were classified as intractable apart from complete recanalization and significant FFS in the current study. An interesting finding is that intractability was not more common in TTOs in the current study. Failure of primary reperfusion modalities may not always result from ICAS-related occlusion (35, 36). Factors such as organization, fibrin-rich thrombi (37), longer thrombus length (36), and collateral status (38) are all additional factors potentially associated with reperfusion failure apart from occlusive etiology. Such factors should be also considered when occlusions refractory to EVT are encountered.

Interrater reliability appeared to also differ among vascular beds. The interrater reliability was very high in the VBA (0.993) and intracranial ICA (0.929) but relatively low in the MCA M1 (0.727). In the MCA, a number of variant forms can exist, such as trifurcation patterns, large anterior temporal artery patterns, etc. Furthermore, there are variations in the length of the M1 segment itself (39). Such variations, and the frequent distal bifurcations masquerading as M1 bifurcations may have led to the lower interrater reliability. In the ICA and VBA, the anatomical morphology and location of the bifurcation is more straightforward, and likely contributed to the higher reliability. In the ICA, hypoplasia of the A1 and A1 occlusion can sometimes be difficult to differentiate, resulting in disagreement. In the VBA, a dominant fetal type posterior cerebral artery with hypoplastic P1 could result in some disagreement. However, the incidences of such disagreement were limited.

While CTA analysis of occlusion types is very practical for early identification of ICAS-related occlusions, our analysis shows that it is limited by a moderate overall sensitivity; a substantial number of FFS did not show TTO. Accordingly, a more sensitive marker may further aid prediction. In this regard, other predictive imaging parameters deserve attention (data outlined in Table 4). Among these, methods that use magnetic resonance imaging (MRI)-based visualization of the thrombus burden or red blood cell-dominancy through gradient echo sequences have been reported (40). Such hypointense signal changes, referred to as susceptibility vessel signs (41), have been shown to be associated with cardioembolic stroke (42). In consecutive MCA occlusion patients, a “negative susceptibility vessel sign” was shown to be a sensitive marker of predicting underlying atherosclerotic stenosis with a high negative predictive value (23). However, the predictive power of negative susceptibility vessel signs were somewhat different in another study (24), showing moderate sensitivity and a lack of statistical power in identifying ICAS-related occlusion. Other studies have also reported the presence of susceptibility vessel signs in stroke due to cerebral atherosclerosis (42). While MRI-based parameters may allow visualization of thrombus burden, treatment delay in door-to-reperfusion time should be reduced (43). Recently, a dynamic CTA-based imaging parameter, the “multisegment clot sign,” was reported to be significantly associated with cardioembolism in patients who received thrombolytic therapy with or without EVT (32). In this report, the absence of the multisegment clot sign was highly sensitive for large artery atherosclerosis, with a modest positive predictive value. While more rapid and precise identification methods should be sought, such parameters may be used in the future to supplement occlusion types in the preprocedural diagnosis of ICAS-related occlusion.

There are some considerations worth mentioning in the current study. First, the comparison between occlusion types and FFS performed in this study should be interpreted with caution, because the best determination method of occlusion type utilizes stent retriever deployment during DSA (25). However, our primary focus was to evaluate occlusion type as a preprocedural surrogate, and analyzed the CTA-determined occlusion type. Because contact aspiration thrombectomy was performed in many cases (*n* = 301, 59.1%), we could not perform DSA-based TTO analysis to support our findings. Likewise, the methods of MIP reconstruction differs from the original reports (26), and also differs from center to center. Differences in MIP slice thickness may change a TTO to BSO, or vice versa. Accordingly, we cannot be sure that our CTA based occlusion type readings may be highly predictive of DSA based occlusion types, and this may be a potential limitation of this study. Second, due to the retrospective nature of this study and changes in CT imaging protocols, not all images could be clearly dichotomized to TTOs or BSOs. Nevertheless, only 16/525 (3.0%) of the total population were classified as inconclusive, showing that occlusion type analysis is applicable in most cases. Third, advances in CT protocols may change one occlusion type to another. In this study, CTA-enhanced images + reconstruction images without MIP images were used in a portion of patients, and may have resulted in a less sensitive analysis, while multiphase CT was used in a number of patients in the later years of the study. Protocols such as multiphase or dynamic CTA can acquire images at later phases after contrast injection, possibly resulting in delayed filling of bifurcation portions distal to the occlusive portion. In theory, such changes might affect the classification between TTO and BSO (26), resulting in a more accurate visualization of thrombus burden (44). Thus, the predictive values reported in this study are liable to changing in future studies, likely increasing the predictive ability of CTA-based occlusion type analysis. Fourth, some patients with TTO in the intracranial ICA with underlying intracranial atherosclerosis might not have been included in our registry because their MCA and ACA were spared; consequently, their initial neurological condition would not be sufficiently severe for EVT to be indicated. Therefore, the findings in the intracranial ICA group should be interpreted with particular caution.

In conclusion, we examined whether the agreement between preprocedural identification of CTA-based TTO and postprocedural identification of FFS may differ among occlusion sites. The identification of TTO in the VBA appeared to have high interrater agreement and the highest agreement with FFS. TTO in the MCA M1 showed relatively low interrater agreement but relatively high agreement with FFS. As for TTO in the intracranial ICA, while interrater agreement was high, the agreement with FFS was low. We believe that our results can aid the selection of patients for prospective randomized trials to address ICAS-related occlusion in the near future.

## Author Contributions

S-JL and JSL contributed to the conception and design of the study, acquisition and analysis of data, and preparation of the manuscript. Y-HH, S-IS, JMH, JWC, D-HK, Y-WK, Y-SK, J-HH, JY, and C-HK contributed to acquisition and analysis of data.

### Conflict of Interest Statement

The authors declare that the research was conducted in the absence of any commercial or financial relationships that could be construed as a potential conflict of interest.
